# Developing a practical approach to assess the older adult psychiatric inpatient’s readiness for an exercise programme

**DOI:** 10.1192/j.eurpsy.2026.11581

**Published:** 2026-07-31

**Authors:** C. I. Ezegwui, S. Gummalla, S. Mathew, W. Goodland, S. De Souza

**Affiliations:** Somerset NHS Foundation Trust, Taunton, United Kingdom

## Abstract

**Introduction:**

Physical exercise can be helpful for a range of psychiatric conditions. In the inpatient environment offering exercise can be difficult, particularly for detained patients who are at risk of absconsion. Indoor gymnasiums build into the hospital can offer a useful opportunity for exercise.

It is commonly advised that before patient’s start an exercise programme that they consult a physician. In the inpatient setting this involves a resident doctor reviewing the patient. However, this raises the question “how should resident doctors assess the older adult inpatient’s readiness for a new exercise programme?”

**Objectives:**

To develop a practical approach to older adult inpatients who want to start a new exercise programme

**Methods:**

A group of resident doctors reviewed the literature and guidance in order to develop a pre-exercise assessment tool. This literature was then amalgamated through discussion to develop a flow chart of assessments to be undertaken prior to using the gym.

**Results:**

The assessment brings together a range of tools that look at the cardiac, respiratory and musculoskeletal health to assess the patient’s readiness for a new exercise programme. If the tool identified significant barrier to exercise then as an integrated mental health and acute trust we can access specialist advice on a case by case basis.

**Conclusions:**

We have created a practical and easy to use approach which can be used in any mental health inpatient setting to ensure that patients are fit to engage in a new exercise programme.

**Disclosure of Interest:**

None Declared

